# Scalable slot-die coated flexible supercapacitors from upcycled PET face shields†

**DOI:** 10.1039/d2ra06809e

**Published:** 2024-04-19

**Authors:** Kiran Kumar Reddy Reddygunta, Andrew Callander, Lidija Šiller, Karen Faulds, Leonard Berlouis, Aruna Ivaturi

**Affiliations:** a Smart Materials Research and Device Technology (SMaRDT) Group, Department of Pure and Applied Chemistry, University of Strathclyde Thomas Graham Building Glasgow G1 1XL UK aruna.ivaturi@strath.ac.uk; b Centre for Molecular Nanometrology, Department of Pure and Applied Chemistry, University of Strathclyde, Technology Innovation Centre 99 George Street Glasgow G1 1RD UK; c Newcastle University, School of Engineering Newcastle upon Tyne NE1 7RU UK

## Abstract

Upcycling Covid19 plastic waste into valuable carbonaceous materials for energy storage applications is a sustainable and green approach to minimize the burden of waste plastic on the environment. Herein, we developed a facile single step activation technique for producing activated carbon consisting of spherical flower like carbon nanosheets and amorphous porous flakes from used PET [poly(ethylene terephthalate)] face shields for supercapacitor applications. The as-obtained activated carbon exhibited a high specific surface area of 1571 m^2^ g^−1^ and pore volume of 1.64 cm^3^ g^−1^. The specific capacitance of these carbon nanostructure-coated stainless steel electrodes reached 228.2 F g^−1^ at 1 A g^−1^ current density with excellent charge transport features and good rate capability in 1 M Na_2_SO_4_ aqueous electrolyte. We explored the slot-die coating technique for large-area coatings of flexible high-performance activated carbon electrodes with special emphasis on optimizing binder concentration. Significant improvement in electrochemical performance was achieved for the electrodes with 15 wt% Nafion concentration. The flexible supercapacitors fabricated using these electrodes showed high energy and power density of 21.8 W h kg^−1^ and 20 600 W kg^−1^ respectively, and retained 96.2% of the initial capacitance after 10 000 cycles at 2 A g^−1^ current density. The present study provides a promising sustainable approach for upcycling PET plastic waste for large area printable supercapacitors.

## Abstract 2

This study reports fabrication of flexible supercapacitor with slot-die coated activated carbon electrodes derived from upcycled PET face shield, displaying excellent energy and power density of 21.8 W h kg^−1^ and 20 600 W kg^−1^ with good flexibility.

## Graphical abstract

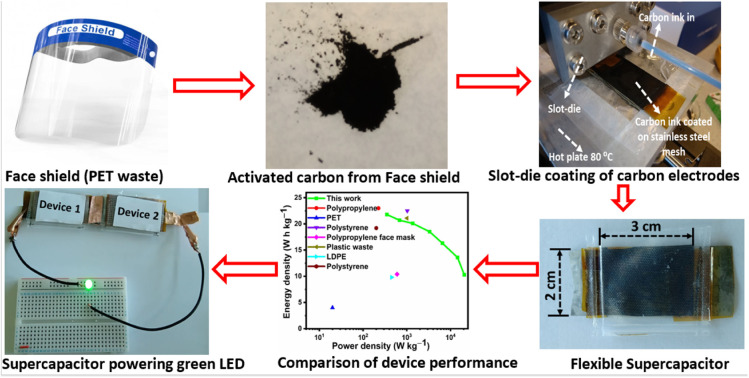

## 1. Introduction

The COVID19 pandemic was a global health emergency as declared by the World Health Organization (WHO) in January 30, 2020.^1^ Governments across the world developed various strategies for containing the virus spread and to reduce the loss of life. One such important measure was use of personal protective equipment (PPE) such as wearing of face masks and face shields during travel and in crowded places.^2^ Although disposable face masks and face shields are primarily designed to protect the health care workers from occupational hazards, authorities had enforced their use by the general public to curb the virus spread.^3^ As a result, large amount of PPEs were produced and used in the last four years which exacerbated the increase in contagious plastic wastes, posing a serious threat to the health, economy and environment. These threats are particularly severe in underdeveloped and developing countries where there are less sophisticated systems for disposing of contagious plastic wastes.

In the last two years of the COVID19 pandemic, face shields were widely used not only by healthcare workers, but also those working at places where the spread of infection was high, such as service counters, receptions, airports *etc.* Face shields are designed to prevent the particles and splashes of fluids that have the potential to transmit the disease from entering the nose, eyes and mouth. Face shields are generally made of polymers such as polycarbonate (PC), polyvinylchloride (PVC), or polyethylene terephthalate (PET).^4,5^ Currently, most of the plastic waste is disposed in three ways: landfill, recycling and incineration. Landfill causes secondary pollution of the land since none of these polymers are biodegradable and incineration releases poisonous gases that pollute the air, thus these techniques are undesirable. Recycling (mechanical and thermo-chemical recycling) is an effective way to reduce plastic wastes by converting them into value added products, leading to sustainable development.^6^ Although large amounts of plastics can be reused through mechanical recycling, additional disinfection protocols are required for contaminated plastic wastes (face shields) before mechanical recycling. Hence thermo-chemical treatment of plastic waste has become the focus of research as it require limited pre-treatment (disinfection) and provides useful carbon materials that can be used for energy storage and other applications.^7,8^

To date, several studies have been reported for the recycling of plastic waste into highly valued products such as carbon nanomaterials for applications spanning from CO_2_ capture, as adsorbents for natural gas storage, for water treatments *etc.*^9,10^ In the present study we explore upcycling the face-shield plastic derived activated carbon nanomaterial in supercapacitors. Supercapacitors possessing high energy and power densities, longer cycle life and wide operating voltage windows are considered to be the next generation energy storage systems that have the potential to replace batteries.^11,12^ They are categorized into electric double layer capacitors (EDLCs) and pseudocapacitors based on their charge storage mechanism. The charge storage in EDLCs is accompanied by the adsorption/desorption of ions at the electrode/electrolyte interface and the capacity is determined primarily on the specific surface area (SSA) of the solid electrode, in contact with the electrolyte ions.^13,14^ On the other hand, pseudocapacitors store the charge in the form of fast and reversible redox reactions occurring at the surface of the electrode as well as *via* the charge storage mechanism of EDLCs. EDLCs based on carbon nanomaterials, such as activated carbon (AC), carbon nanotubes (CNTs), carbon nanofibers (CNFs) and graphene, have been widely reported in the literature in the past two decades.^15^ Although wide spread research has been reported on graphene and CNTs based supercapacitors, these materials are highly priced and involve tedious synthesis processes. With high electrical conductivity, tunable surface area and pore volume, excellent electrochemical stability, cost effective synthesis and eco-friendly nature, activated carbon has emerged as a prominent electrode material for supercapacitor application.^16,17^ For “waste-to-energy” conversion, activated carbon from agro, animal, plastic and industrial waste precursors has been successfully developed using pyrolysis techniques and has been explored as an electrode material for supercapacitors.^18–20^ Hence, it is promising to convert COVID19 plastic waste into carbonaceous materials for energy storage application, which not only nullifies the environmental threat posed by them, but also presents a sustainable way to produce advanced electrode materials for supercapacitor applications. In this regard, in the present study we devise a single stage approach for the conversion of used PET face shields into activated carbon.

As noted above, supercapacitors exhibit high energy densities as well as high power densities with a wide range of applications from consumer electronics to hybrid automobiles. Manufacturing-friendly scalable coating methods are required for the successful transition of lab-scale research on activated carbon-based supercapacitors to become a viable commercial product. Electrode preparation is the first step in the fabrication of supercapacitors. Each electrode is made up of an electroactive material that is either directly coated onto a current collector or is bonded *via* an electrically conductive adhesive layer.^21,22^ At the laboratory scale, researchers mostly focus on small-area devices, and large area supercapacitors with carbon electrodes are currently difficult to produce because the performance typically degrades significantly as the surface area is increased. Among the coating technologies, processes such as spray coating,^23–25^ electrospinning,^26^ 3D printing,^27,28^ gravure printing,^29,30^ doctor-blading,^31,32^ and slot-die coating^33^*etc.*, have been explored to coat supercapacitor electrodes. But each of these fabrication techniques have their own advantages and limitations. For example, spray coating is suitable for large-area coating, but the operating person must be careful as large amount of sprayed particles and dust are produced, requiring the operator to wear suitable breathing apparatus and protective clothing. Moreover, the sprayed particles and dust are easy to fly and deposit on surrounding equipment in the lab which necessitates protective sealing. Moreover, preparing carbon based flexible electrodes with spray coating requires bulk amount of material and also the adhesion and durability of the coated films is a challenge. On the other hand, electrospinning is a scalable, simple and effective manufacturing technique to produce flexible nanofibers *via* applying a high voltage to a polymer solution.^34^ Carbon nanofibers (CNFs) with diameters ranging from sub-nanometers to several micrometers, have brought synergic advantages of excellent flexibility and relatively high specific surface areas. But the CNF electrodes prepared with electrospinning technique suffer from low specific capacitance, low energy density and poor cyclic stability which is a serious drawback for preparing flexible carbon electrodes with electrospinning process.^35^ Similarly, the doctor-blade coating is a commonly used method in laboratories for making supercapacitor electrodes on large area current collectors. But doctor blading is suitable to create thicker films as it requires large amount of material to prepare a slurry. We believe that slot-die coating has the possibility to overcome the above-mentioned limitations. Slot-die coating is one of the prevailing technologies in manufacturing lithium ion secondary battery electrodes,^36–38^ solar cell,^39,40^ light-emitting diodes,^41,42^ fuel cells^43^*etc.* The major advantage of combining activated carbon with slot-die coating is that it is scalable which means the process would work on an industrial scale. Moreover, this method is adaptable, allowing it to work with high or low viscosity solutions and can be used to deposit films with wide range of thicknesses. Also, slot-die coating involves less wastage as the flow of solution is very well controlled quick process and doesn't require additional heating stage to dry the films. To the best of our knowledge, there is only one report on the use of slot-die coating for supercapacitor electrode fabrication. One possible reason that hinders the application of slot-die coating in supercapacitors is the ink formulation (solvent used and binder concentration) because certain dry film properties such as thickness, homogeneity, composition, microstructure, coated-edge alignment, and front-side-to-back-side alignment (for double-sided electrodes) are determined by a balance between the electrode materials ink formulation and the coating parameters used. These parameters play a crucial role in determining the electrochemical performance of supercapacitors. As a result, optimising coating parameters and ink formulations to manufacture high-performance carbon electrodes for supercapacitors is important, as slot-die coating technology is a potential technique for scale-up of supercapacitor production and commercialisation.

Apart from coating parameters, the other crucial factors that affect the electrochemical performance of coated carbon electrodes are the type of binder and its concentration. Several studies centred on the design and development of activated carbon as a supercapacitor electrode material have been reported^44–47^ but none have explored optimising the impact of binder concentration. The binder is a crucial component that allows for (i) mechanical strength of the coated electrode films and (ii) good adherence *via* optimal electrical contact between the porous electroactive material and the current collector.^48,49^ Different electrode preparation protocols have been reported for supercapacitor studies, and these different methodologies have a significant impact on supercapacitor performance since the morphology of porous carbon materials can be altered during electrode preparation. PVDF (polyvinylidene difluoride) and PTFE (polytetrafluoroethylene) are the most extensively used binders. However, PVDF is electrically insulating and it is usually treated with the hazardous expensive organic solvent, *N*-methyl-2-pyrrolidone (NMP) during electrode fabrication. PTFE can be processed as a dispersion in water without any organic solvent which makes it a potential alternative to manufacture electrodes for energy storage applications. But the spherical shaped PTFE particles reduce the interfacial contact area because of their particle size (≈100–200 nm) which blocks the electrochemically active surface of the porous materials to the electrolyte.^50^ Even-though PVDF and PTFE are widely used binders, they suffer from poor binding affinity and tend to agglomerate when used in ink formulation.^49,50^ On the other hand, Nafion is an amphiphilic molecule with excellent ionic conductivity. It also prevents agglomeration of the carbon particles and enhances the interfacial wettability between electrode and electrolytes, allowing for faster ion transport.^51,52^ In the present work, Nafion was chosen as the binder for formulation of the activated carbon ink and the effect of its concentration on the electrochemical performance of overall device was studied.

In this investigation, high surface area activated carbon was prepared by single stage activation of upcycled PET face shield and tested for supercapacitor electrode applications. The textural characteristics, composition and morphology of the synthesized activated carbon have been carefully evaluated using different analytical techniques. This study demonstrates a straightforward, safe and effective approach to address disposal of PET plastic waste from used face shields. Additionally, the promising characteristics of the produced porous carbon materials for electrochemical supercapacitor applications makes it even more enticing. Scalable slot-die coating has been explored to fabricate large area supercapacitor electrodes by coating activated carbon ink onto stainless-steel mesh current collectors. The key parameters such as binder concentration and number of coatings have been systematically optimized to obtain good electrode coating and high-performance supercapacitors. The supercapacitors using slot-die coated electrodes with 15 wt% Nafion concentration delivered the highest energy density of 21.8 W h kg^−1^ at 348.8 W kg^−1^ power density with excellent cyclic stability of 96.2% capacitance retention after 10 000 cycles, which is comparable to or better than the carbon electrodes fabricated *via* conventional coating.^53–55^ This study presents a sustainable approach of upcycling PET plastic waste into high-value products. Furthermore, a low-cost, scalable slot-die coating method for fabricating high-performance supercapacitor carbon electrodes, has been demonstrated.

## 2. Experimental section

### 2.1 Materials

The used PET face shields were employed as the precursor material for synthesising activated carbon. Potassium hydroxide (KOH), sodium sulfate (Na_2_SO_4_), Nafion binder, 1-methylpyrrolidine, hydroxyethyl cellulose (HEC) were obtained from Merck. Isopropanol was purchased from VWR chemicals. Hydrochloric acid was obtained from Fisher Scientific. All chemicals were used without further purification.

### 2.2 Single stage activation of face shield into activated carbon

The synthesis of porous activated carbon from waste PET face shields was carried out as follows: 5.2 g of shredded face shield pieces and 10.4 g of KOH were mixed in 25 ml DI water under mechanical stirring at 60 °C for 1 h. The mixture was then dried at 110 °C inside a hot air oven overnight. Upon drying, the transparent face shield pieces were transformed into solid white product. The solid white product was then transferred into a ceramic boat and then activated at 700–900 °C for 1 h inside a tube furnace under N_2_ atmosphere. Finally, the activated products were washed with 1 M HCl and DI water several times (until a neutral pH was achieved) and then dried at 110 °C overnight. The as-synthesized samples are termed as ACF-*T* in the following sections, where *T* corresponds to the activation temperature.

### 2.3 Preparation of ACF-*T* electrodes for electrochemical testing

Working electrodes were designed from activated carbon derived from upcycled waste face shield by coating a slurry (∼2 mg) consisting of 90% of as-prepared carbon sample (active material), 10 wt% polyvinylidene fluoride (PVDF) along with a few drops of *N*-methyl-2-pyrrolidone (NMP) on 1 cm × 1 cm stainless steel mesh electrodes, followed by drying at 80 °C for 2 h.

### 2.4 Slot-die coating of electrodes and device assembly

**Fig. 1:**
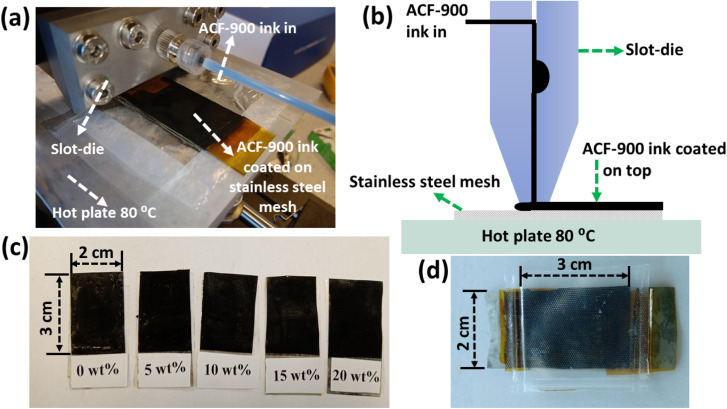
(a) Photograph of slot-die coater head during coating on stainless steel mesh current collector. (b) Schematic of slot-die coating. (c) Photographs of slot-die coated electrodes with different concentrations of Nafion. (d) Photograph of assembled flexible supercapacitor.

Slot-die coater is a device that can deposit inks while also uniformly spreading and establishing coating breadth. The ink flow chamber is housed inside the steel body of the die. To prepare the ACF-900 ink, 20 mg of ACF-900 was mixed with different amounts of Nafion (0 to 20 wt%) dispersed in IPA and sonicated for 4 h. The activated carbon (ACF-900) film was coated on top of stainless-steel mesh (length, width and thickness of 4.5 cm, 2 cm and 0.07 mm, respectively) current collector by using the scalable slot-die coating technique. Fig. 1(a) and (b) shows a photograph and schematic of the slot-die coater head and the coated stainless-steel mesh electrode. 5 ml of ACF-900 ink was loaded into a syringe and assembled with the slot-die facing the current collector placed on the hot plate. The hot plate was pre-set to 80 °C before initializing the coating. After selecting the coating parameters (listed below), the process was initiated and a carbon film was coated on top of the current collector (on an area of 3 cm × 2 cm). After each coating, the mesh electrode was allowed to dry (on the hot plate pre-set at 80 °C) for 10 min before successive coatings were applied. The process was repeated 5 times so that a homogenous film was formed on top of the current collector. The coated carbon electrodes were then dried on the hot-plate and trimmed to the required size for device fabrication. The photographs of slot-die coated electrodes with different Nafion concentrations (wt%) are shown in Fig. 1(c) and the assembled flexible supercapacitor is shown in Fig. 1(d).

Parameters used for slot-die coating:

Number of shams used = 2

Coating length = 30 mm

Coating speed = 2.09 mm s^−1^

Dispense rate = 1.069 μl s^−1^

Stage delay = 3.00 s

Temperature = 80 °C

Drying time = 10 min for each coating

Note: The coating parameters employed here are for the specific solvent and binder combination (IPA + Nafion) used in this study for ink formulation. These coating parameters may vary for different solvents and binders.

The device was finally assembled by sandwiching the slot-die coated carbon electrodes and a gel-polymer electrolyte. For the latter, hydroxyethyl cellulose (HEC) was chosen as a polymer host and sodium sulfate (Na_2_SO_4_) was used as the ionic salt to improve the ionic conductivity of the gel electrolyte. The HEC/Na_2_SO_4_ gel electrolyte was prepared according to our previous published work.^56^ Typically, 2 g of HEC was dissolved in 20 ml of DI water at 90 °C and stirred for 1 h. Next, 2 g of Na_2_SO_4_ dissolved in 10 ml of DI water was added dropwise for 15 min to the solution and stirred at 90 °C for another 1 h to produce a transparent HEC/Na_2_SO_4_ gel electrolyte. Before assembling the flexible supercapacitor device, the HEC/Na_2_SO_4_ gel electrolyte was smeared on the surface of the 3 cm × 2 cm electrodes and allowed to dry overnight in a fume hood at room temperature. A thin white coloured layer of HEC/Na_2_SO_4_ was formed on the surface of both electrodes. The electrodes with the gel layer on the top were then carefully sandwiched without any separator and sealed tightly with a vacuum sealer (soft vacuum mode). To improve the contact between the electrode and electrolyte interface and to completely seal the device, a pellet press machine was used to apply 1 ton of pressure for 5 minutes.

### 2.5 Material characterization

The specific surface area and pore volume of the prepared carbon samples were analysed *via* Brunauer–Emmett–Teller (BET) and Non-Linear-Density-Functional-theory (NLDFT) method using Micrometrics ASAP 2020 porosity analyser at 77 K. The crystallographic nature of the samples was examined by X-ray diffraction (XRD) using a Bruker D2 Phaser system utilising monochromatic CuKα radiation with a wavelength of 1.5406 Å. The samples were scanned in the range 5–80° with an increment of 0.04 on the 2*θ* scale. The substrates were set to a rotation speed of 8 per min throughout the measurements. The morphological features were obtained using FEI Quanta 250 FEGSEM, with a 5 kV electron beam. Transmission electroscope microscope (TEM) images were obtained using a JOEL 2100F FEG TEM operated with an accelerating voltage of 200 kV. X-ray photoemission spectroscopy (XPS) analysis was conducted using a Thermo Scientific K-alpha X-ray Photoelectron Spectrometer™ (Thermo Scientific, East Grinstead, UK). High resolution photoemission spectra of specific element regions (C 1s, O 1s) were collected at 40 eV pass energy of hemispherical electron analyser with 0.05 eV energy step size. Spectra were acquired using a monochromatic Al Kα X-ray source with an output energy of 1486.6 eV with a maximum X-ray beam spot size of 400 μm. Surface charge compensation was obtained with a low energy dual-beam electron/ion flood gun. To study the defective/graphitic nature of the as-prepared samples, Raman spectra were measured using a WiTec Raman microscope fitted with 10× objective, with a 2.03 mW of power at the sample surface, a 532 nm excitation wavelength and 10 s acquisition time. Fifteen spectra were obtained of each sample, and the resultant spectra were subsequently baselined and averaged using MATLAB. The Raman and XPS spectra of all the samples were deconvoluted using Fityk software using Voigt fitting.

### 2.6 Electrochemical measurements

The electrochemical performance of all the as-synthesized activated carbon samples (from upcycled PET face shields) coated on stainless steel current collectors was investigated in three and two electrode configurations using an Autolab PGSTAT 302N system controlled by Nova 2.1.5 software. A Ag/AgCl reference electrode was employed with a platinum rod as the counter electrode. 1 M Na_2_SO_4_ aqueous solution was used as the electrolyte in the three-electrode configuration and the HEC/Na_2_SO_4_ gel electrolyte in the two-electrode configuration. Cyclic Voltammetry (CV) and Galvanostatic Charge–Discharge (GCD) analysis were carried out within a 1 V potential window (Δ*V*) between −0.2 and 0.8 V. Electrochemical Impedance Spectroscopy (EIS) was recorded for all the electrodes under an AC perturbation of 5 mV within 10 mHz to 100 kHz frequency range at the open circuit potential (OCP).

From the charge–discharge measurements, the specific capacitance of the working electrode can be calculated using the formula (1).^57^1
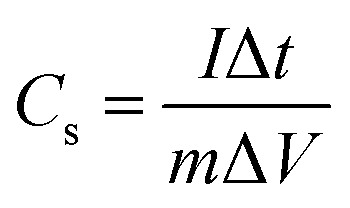
where *I* is the applied current (A), Δ*t* is the discharge time (s), Δ*V* voltage window (V), and *m* is the mass load of active material on the mesh electrode (mg).

The flexible supercapacitor device was subjected to electrochemical tests, using the two-electrode configuration with HEC/Na_2_SO_4_ electrolyte to evaluate its performance. The performance evaluation parameters, electrode specific capacitance *C*_es_ (F g^−1^), total capacitance (*C*_t_) of the device, energy density *E* (W h kg^−1^) and power density *P* (W kg^−1^), were estimated from galvanostatic charge–discharge measurements in a two-electrode configuration by means of the following equations.^58,59^2
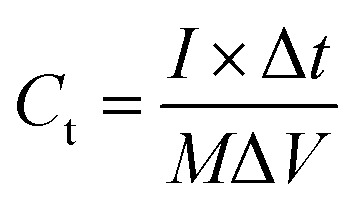

3 *C*_es_ = 4 × *C*_t_

4
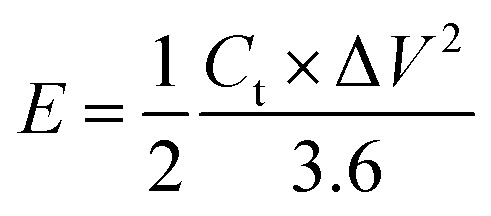
5
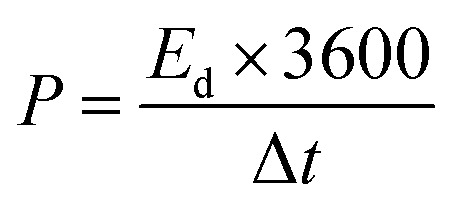
where *I* represent the current (in ampere), Δ*t* is the discharge time, *M* is the total weight of active material within the two electrodes and Δ*V* is the potential window.

## 3. Results and discussions

In this study, a single stage potassium bicarbonate (KOH) activation was used for the synthesis of high surface area porous activated carbon from used face shield (PET). Before the activation process was performed, the thermal degradation of face shield (PET) pieces was characterized using thermogravimetric analysis (TGA). According to the TGA measurements shown in Fig. S1(a),† the face shield (PET) pieces showed two stage decomposition process. PET is stable till 300 °C and the actual decomposition is initiated above 300 °C and spanned over long period till 750 °C. Sharp weight loss of ∼72% occurred between 300 to 420 °C attributed to the rapid pyrolysis of PET. Weight loss of ∼18% occurred between 420 to 750 °C. The initial decomposition of PET is mainly due to the presence of volatile impurities like additives used to bond the polymer chains during the preparation process. The second degradation step occurs in between 420 to 750 °C, which is attributed to the degradation of thermally stable cross-linked carbonaceous structures formed during the first degradation step.^60–62^ After 750 °C, a constant decomposition is observed which indicates the formation of solid carbon materials. Hence, based on TGA result, we chose the starting temperature of 700 °C. But, in the literature it has been reported that carbonaceous materials with high specific surface area and pore volume can be produced when the activation process is carried out at temperatures higher than 700 °C. Thus, in the current study, the potassium hydroxide (KOH) activation was carried out at three different temperatures: 700 °C, 800 °C and 900 °C.^63^

### 3.1 Structural and textural characterization

**Fig. 2:**
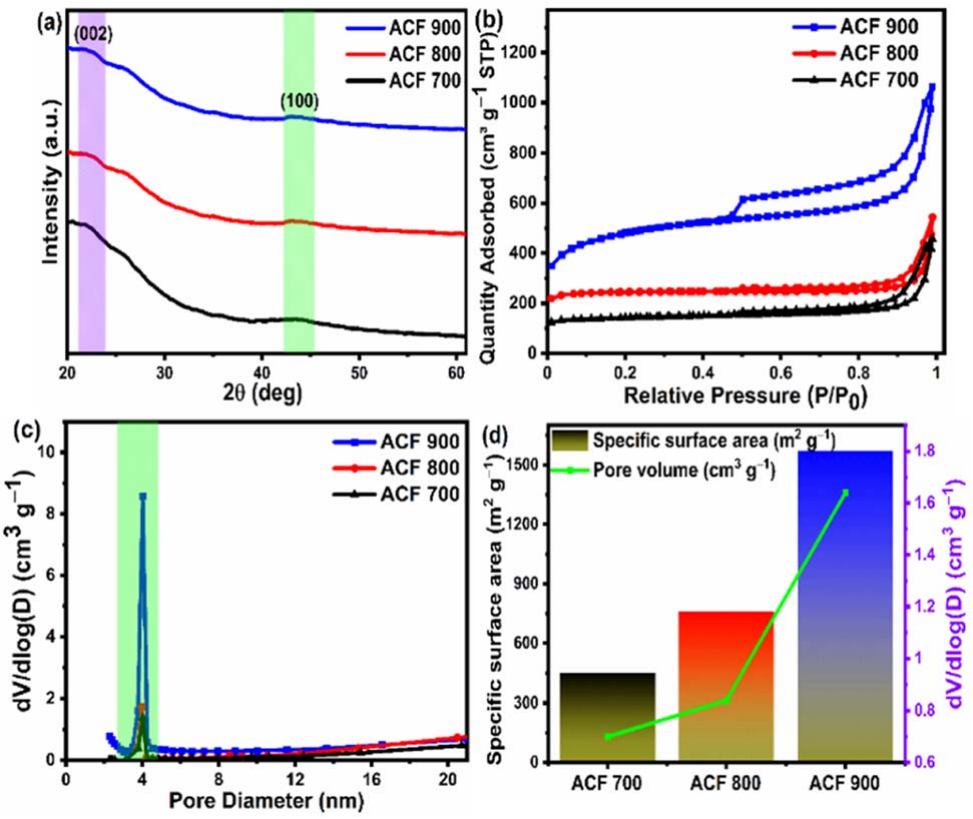
(a) XRD patterns of activated carbon derived from upcycled waste face shield (ACF-*T*) activated at different temperatures (*T* = 700 °C, 800 °C and 900 °C). (b) The N_2_ adsorption/desorption isotherms (c) pore size distribution data. (d) Graph showing specific surface area and pore volume of ACF*T* samples obtained from upcycled waste face shield activated at different temperatures.

The XRD patterns of the face shield derived activated carbon [ACF-*T*] samples activated at three different temperatures are shown in Fig. 2(a). Similar XRD profiles were observed for all these samples with diffraction peaks at 2*θ* = 23° corresponding to the (002) plane of amorphous carbon and the low intensity diffraction peak around 43° corresponds to (100) planes of graphitic carbon.^64,65^ Moreover, the intensity of the peak at ∼43° is weak compared to the peaks at 23° suggesting that the crystallites in the ACF-*T* possess intermediate structures with a dominant amorphous nature and weak graphitic state which can also be termed as random layer lattice structures.^66,67^ In the XRD spectrum of the carbon samples, there is also a broad peak at 2*θ* = 26.1°, indicating the presence of graphitic carbon embedded in amorphous carbon.^68^

The porosity of the ACF-*T* samples was measured using N_2_ adsorption/desorption isotherms and pore size distribution plots as shown in Fig. 2(b) and (c). The isotherms of all the ACF-*T* samples belong to a combination of type I and type IV isotherms with hysteresis behaviour (based on IUPAC classification) indicating the prepared carbon possess micro and meso porous framework.^69,70^ The specific surface area and pore volume of all the ACF-*T* samples are shown in Fig. 2(d). With the increase in activation temperature from 700 to 900 °C (*P*/*P*_0_ < 0.1), the quantity of N_2_ adsorbed at low relative pressures increases considerably indicating higher micro porosity in the ACF-900 sample. Moreover, the ACF-900 sample shows a dominant H3 type hysteresis loop within the intermediate pressure section (0.4 < *P*/*P*_0_ < 1) indicating the sample consists of abundant mesopores.^71^ From Fig. 2(d) it can be inferred that the ACF-900 sample possesses the highest specific surface area (1571.4 m^2^ g^−1^) and highest pore volume (1.64 cm^3^ g^−1^) and that the adsorption volume of the ACF-*T* samples increases with activation temperature. The enhancement of the pore volume is particularly beneficial for EDLC based supercapacitor as the capacitance is directly proportional to the specific surface area. Also, the pore size distribution of ACF-*T* samples was obtained using the NLDFT method as shown in Fig. 2(c), which indicates that the pore size of all the samples is mainly concentrated in the 3–5 nm range, confirming the presence of abundant mesopores. The unique pore characteristics combined with high SSA and pore volume (1.64 cm^3^ g^−1^) in ACF-900 sample leads to a larger electrode/electrolyte interface for charge–discharge.

**Fig. 3:**
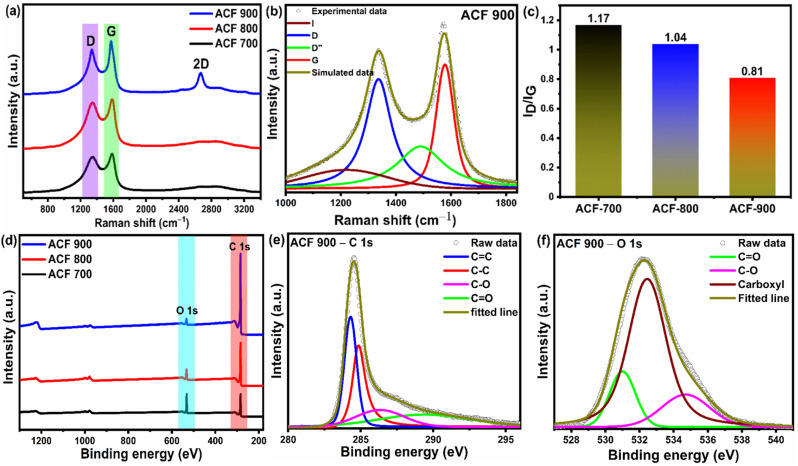
(a) Raman spectra of different ACF-*T* samples obtained from upcycled waste PET face shield measured using 532 nm laser excitation, 2.03 mW laser and a 10 s acquisition time. (b) Deconvoluted Raman spectra of ACF900 sample performed using Fityk software. (c) Graph showing the degree of graphitization (*I*_D_/*I*_G_) of ACF-*T* samples obtained at different activation temperatures. (d) XPS survey spectra of all ACF-*T* samples. (e) Deconvoluted C 1s spectra of ACF900 sample. (f) Deconvoluted O 1s spectra of ACF-900 sample.

Further investigations on the microstructure of face shield derived activated carbon were conducted using Raman spectroscopy, shown in Fig. 3(a). Two characteristic peaks at 1340 cm^−1^ and 1577 cm^−1^ are typically visible in the Raman spectra of all ACF-*T* samples, attributed to the D-band and G-band respectively.^72,73^ The D-band is related to the defects in the amorphous carbon framework (sp^3^ type), whereas G-band is associated with the sp^2^ hybridized carbon atoms in the graphitic layer.^73^ Moreover, the 2D band occurring at 2670 cm^−1^ is stronger in ACF-900 compared to the other samples confirming the presence of graphene sheet like structures in ACF-900.^74^ But the intensity of 2D band is much lower than that of the D and G-bands indicating the dominance of the amorphous nature in the overall samples. The Raman spectra of ACF-*T* samples was further deconvoluted into four distinct peaks as shown in Fig. 3(b) and ESI Fig. S2(a) and (b).† ACF-900 has four peaks centred around 1210 ± 5 cm^−1^ (I-band), 1340 ± 5 cm^−1^ (D-band), 1517 ± 5 cm^−1^ (D′′-band) and 1575 ± 15 cm^−1^ (G-band) respectively as shown in Fig. 3(b).^75,76^ The corresponding peak deconvolution parameters are given in the ESI Table S1.† According to the literature, D′′-band represents the polyenes or oligomers whereas I band corresponds to amorphous carbon and distorted graphitic structures in carbon material, D band indicates the disordered structures caused by the defects in the carbon lattice and G-band refers to graphitic carbon (sp^2^ hybridized) respectively. Therefore, the resultant Raman peaks confirm the carbonization process of the recycled face shield. The *I*_D_/*I*_G_ ratio is often studied to quantify the degree of graphitization, the lower the *I*_D_/*I*_G_ ratio, the higher the sp^2^ graphitic microstructure in the obtained sample. From Fig. 3(c), it can be deduced that ACF-900 has a lower *I*_D_/*I*_G_ ratio indicating relatively higher sp^2^ hybridized graphitic carbons compared to ACF-700 and ACF-800 samples.

The chemical composition of ACF-*T* samples were analysed using XPS and the survey scan is shown in Fig. 3(d). High intensity C 1s and O 1s peaks are observed in the survey spectra of the ACF-*T* samples at 284.5 eV and 532 eV, respectively. A sharp increasing trend of C 1s peak is observed with the increasing temperature, indicating a good nature of graphitization. The existence of oxygen in the samples can be attributed to the incomplete activation of the oxygen functional groups in the waste PET and the oxygen absorbed at the surface of the samples. Besides that, the KOH activation could also efficiently introduce O heteroatoms into the carbon framework.^77^ In order to analyse the nature of the heteroatoms present in the as-prepared activated carbon samples, peak deconvolution was performed for ACF-900 as show in Fig. 3(e) and (f). The deconvoluted spectra of ACF-700 and ACF-800 are shown in Fig. S3(a)–(d).† The C 1s spectrum have been fitted with four independent peaks assigned to sp^2^ C=C (284.3 eV), sp^3^ C–C (284.8 eV), C–O (286.3 eV) and C=O (289.4 eV) from the high-resolution elemental spectrum [Fig. 3(e)], while the O 1s spectrum [Fig. 3(f)] can be deconvoluted into three peaks corresponding to C=O (531.6 eV), C–O (533 eV) and carboxyl groups (534.3 eV) respectively.^78,79^ It's worth noting that activated carbon with O functional groups improves the wettability of the carbon surface and provides additional pseudocapacitance to the electrode material.^80^ The chemical compositions for the ACF-*T* samples characterized by XPS were calculated from the core energy levels of individual elements using peak intensities as shown in ESI Table S2.† As illustrated in Table S2,† ACF-900 mainly consisted of carbon (94.64%) accompanied by oxygen (5.36%). Obviously, as the activation temperature rose from 700 °C to 900 °C, oxygen content on the ACF decreased while carbon content increased, moreover, from the deconvoluted C 1s spectra of ACF-*T* samples, it can be observed that sp^2^ C=C (284.3 eV) peak is more dominant in ACF-900 sample as compared to ACF-700 and ACF-800 sample. Therefore, in combination with Raman spectra, the XPS results also confirm the dominant graphitic carbons in ACF-900.

**Fig. 4:**
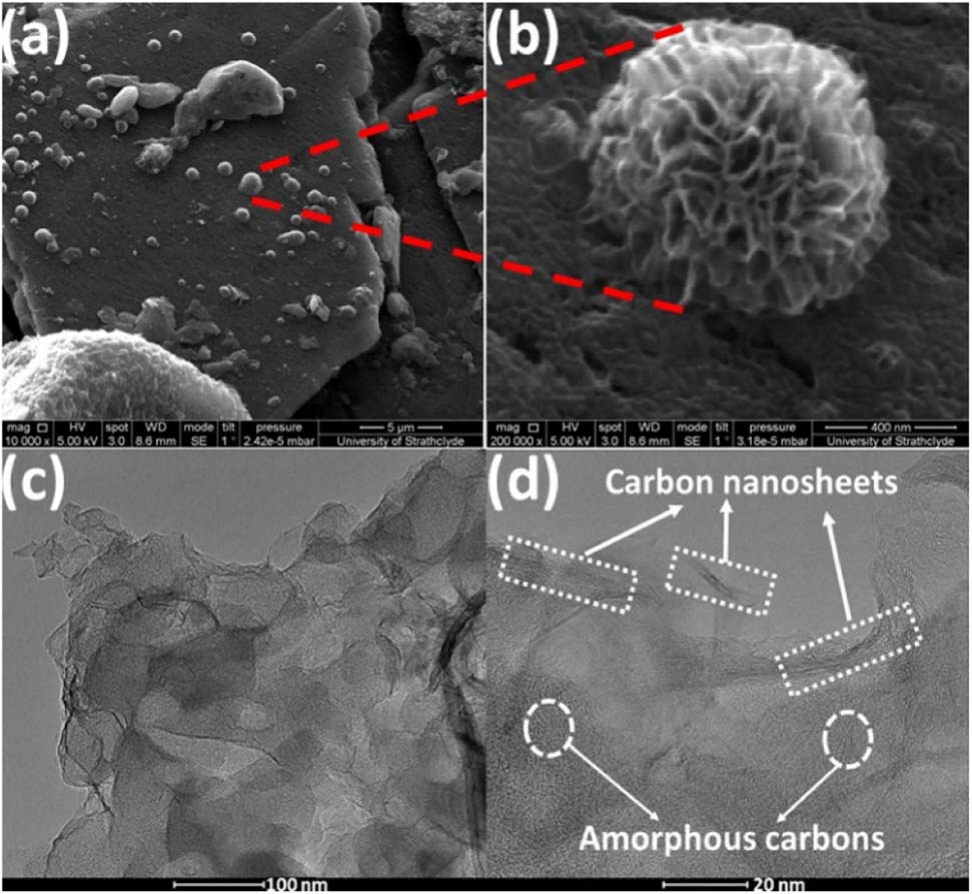
(a) and (b) FESEM images of ACF-900 sample at different magnifications; (c) and (d) HRTEM images of ACF 900 sample at different magnifications.

Fig. 4(a) and (b) shows FESEM images of activated carbon ACF-900 obtained from upcycling waste face shields activated at 900 °C. FESEM images of ACF-700 and ACF-800 samples are shown in the ESI Fig. S4(a)–(d).† FESEM images of all the samples show the presence of sheet like structures but FESEM image of ACF-900 in Fig. 4(a) shows amorphous flaky structures with ultrathin carbon nanosheets accumulated to form spherical flower like structures which are distributed randomly on top of the amorphous porous flakes. The magnified image of ACF-900 shown in Fig. 4(b) clearly shows the spherical flower like structure with interconnected ultrathin carbon nanosheets. The porous-activated carbons are created by KOH activation at high temperature of 900 °C, which created abundant high surface area pores, whereas an increase in temperature from 700 to 900 °C is assumed to accumulate these carbon nanosheets to form spherical flower like structures. HRTEM images of the ACF-900 sample are shown in Fig. 4(c) and (d) which clearly depicts the presence of amorphous carbons with carbon nanosheet framework connecting the high surface area porous carbon. The porous-activated structure with interconnected carbon nanosheets is crucial for electrochemical supercapacitors because the porous structure is responsible for the charge accumulation and the interconnected carbon nanosheets provides efficient ionic transmission in the inner pores of the amorphous carbons.

### 3.2 Electrochemical performance in three electrode

**Fig. 5:**
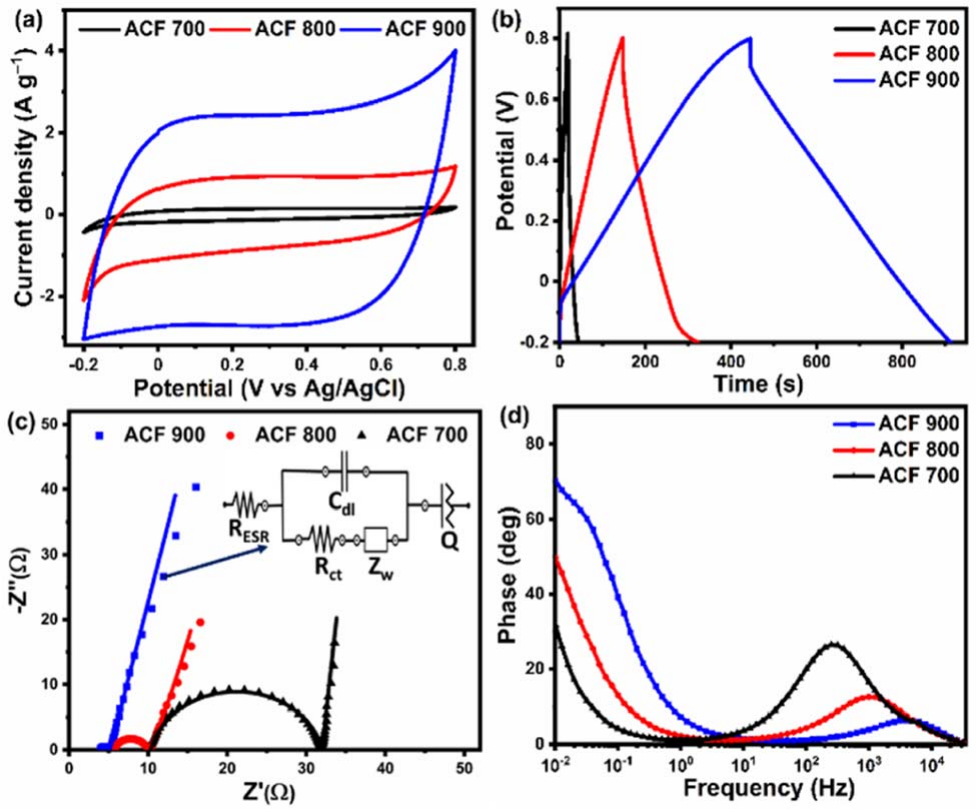
(a) CV curve of different ACF-*T* electrodes within a potential window of −0.2 to 0.8 V at 5 mV s^−1^ scan rate. (b) GCD curves of different ACF-*T* electrodes at 0.5 A g^−1^ current density. (c) Nyquist plot of different ACF-*T* electrodes with inset showing the equivalent circuit fitting of ACF 900 electrode. (d) Phase plot of ACF-*T* electrodes in 1 M Na_2_SO_4_ aqueous electrolyte.

On the basis of structural analysis, the as-prepared ACF-*T* carbon materials obtained from upcycled PET face shield are expected to possess excellent electrochemical characteristics. Using cyclic voltammetry (CV) and galvanostatic charge discharge (GCD) measurements in 1 M Na_2_SO_4_ electrolyte solution (potential range: −0.2 to 0.8 V; sweep rate: 5 to 100 mV s^−1^; current density: 0.5 to 10 A g^−1^), the electrochemical properties of the electrodes were studied [Fig. 5(a) and (b)]. All the ACF-*T* electrodes exhibited the classical ‘rectangular’ shaped voltammogram within the −0.2 to 0.8 V voltage window at 5 mV s^−1^ indicating the highly reversible and capacitive nature of the carbon material, with ACF-900 having the highest current and largest area, implying the highest specific capacitance (and best candidate as electroactive material) for supercapacitors. The GCD curves of the as-prepared samples [Fig. 5(b)] shows triangular shaped charge discharge behaviour, which is typical of carbonaceous materials. Moreover, the ACF-900 electrode exhibited longer charge discharge durations, indicating the ability of ACF-900 to store a large amount of charge. The specific capacitance of ACF-*T* samples, evaluated using eqn (1), indicates that the ACF-900 electrode possesses the largest specific capacitance (228.2 F g^−1^) compared to the other electrodes implying that a greater number of electrolyte ions are able to access the pores of this carbon electrode during the charge–discharge process. Electrochemical impedance spectroscopy (EIS) was also employed to measure the characteristics of the ACF-*T* electrodes in a three-electrode system. The Nyquist and Bode phase charts of the ACF-*T* electrodes measured in the frequency range between 0.01–100 kHz, with AC amplitude of 5 mV are shown in Fig. 5(c) and (d). In the high-frequency area, all samples have a semicircle which corresponds to the charge transfer resistance (*R*_ct_) between electrolyte and electrode. As can be observed from Fig. 5(c), the *R*_ct_ of the ACF 900 is 1.2 Ω, which is lower than the *R*_ct_ of the ACF-700 (21.9 Ω) and ACF-800 (4.2 Ω), implying that the ACF-900 facilitates higher charge transport kinetics within its pores compared to the ACF-700 and ACF-800 electrodes. Furthermore, the equivalent series resistance (*R*_ESR_) of the electrodes is represented by the intersection of Nyquist plots on the real axis. The *R*_ESR_ values of the ACF-700, ACF-800, and ACF-900 are 3.9, 5.6 and 10.3 Ω respectively. According to these measurements, ACF 900 has the smallest *R*_ct_ and lowest *R*_ESR_ which is attributed to its greater surface area and sheet like morphologies. The equivalent circuit corresponding to the impedance spectra of ACF 900 electrode was shown in the inset of Fig. 5(c) and the equivalent circuit model of ACF 700 and ACF 800 are provided in ESI Fig. S5(g) and (h).† The equivalent circuit consists of series resistance (*R*_ESR_), charge transfer resistance (*R*_ct_), Warburg impedance related to the electrolyte ion diffusion (*Z*_w_), double layer capacitance (*C*_dl_) and constant phase element (*Q*). At high frequencies, the *R*_ct_–*C*_dl_ circuit is responsible for the transfer of ions inside the pores of the electroactive material, whereas *Z*_w_ is responsible for the diffusion of electrolyte ions in the middle frequency region. At low frequencies, the inclined straight line indicates the pseudo-capacitive behaviour of the ACF 900 electrode, which is represented by *Q* in the equivalent circuit. From the fitted parameters, the ACF 700 electrode shows high resistance and low capacitance, indicating poor capacitive performance due to deficiency of active surface area and pores to collect electrolytic ions. The series resistance (*R*_ESR_) and charge transfer resistance (*R*_ct_) of the ACF 700 electrode is slightly higher than the ACF 800 and ACF 900 electrode, which is due to the presence of a slightly higher amount of oxygen functionalities in the ACF 700 electrode. Besides, the Bode plots of ACF 700, ACF 800 and ACF 900 electrodes, reveals the dependency of phase angle on frequency. Fig. 5(d) shows the variation of phase angle with respect to frequency. At low frequency (10 mHz), the ACF-900 electrode shows maximum phase angles of −70.9°, representing dominated capacitive behavior of the electrodes, while ACF-700, which has a maximum of −30.8°, attributes the dominated resistive behavior of the electrode. Moreover, the slightly low phase angle of ACF-700 and ACF-800 than the ACF-900 electrode is mainly due to more pseudocapacitive nature of the electrodes. From the Nyquist and Bode phase plots, it can be concluded that ACF-900 has the highest phase angle confirming that it has the best capacitive behaviour and rapid electron transport kinetics. The outstanding electrochemical performance of ACF-900 can be attributed to its high accessible surface area which promotes the ion adsorption/desorption process more effectively. Additionally, the ACF-900's hierarchical porous structure, which has the highest pore volume and nanosheet-like structures provides shorter diffusion paths to the interior surfaces of the porous electrode, accelerating the electron transfer or decreasing the charge transfer resistance (*R*_ct_). Table 1 shows the specific surface area and electrochemical performance of the ACF-900 electrode compared with other activated carbon obtained from different plastic materials reported in the literature.^46,73,81–87^ As shown in Table 1, the ACF-900 coated mesh electrodes showed excellent electrochemical performance in 1 M Na_2_SO_4_ electrolyte and comparable to the values reported in the literature. This could be due to the higher degree of graphitization and specific surface area (SSA) of ACF-900, as evident from Fig. 3(d) and (b). Indeed, the ACF-900 obtained from upcycled waste PET face shield showed comparable or better electrochemical performance in aqueous electrolyte to other plastic waste-derived activated carbon previously reported in the literature. It has been observed that the electrochemical performance of the most reported carbonaceous materials obtained from plastic wastes was found to be dominated by carbon materials with a high surface area which varies depending on the synthesis protocols, catalyst used in the activation process and electrolyte. For example, Tang *et al.*^81^ utilized iron oxide nanoparticles and urea as the catalyst for the degradation of polystyrene waste using a two-step conversion process which resulted in a high surface area of 2110 m^2^ g^−1^ and specific capacitance of 284 F g^−1^. The process employed in that study is rather tedious and requires high amounts of hydrochloric acid to remove the iron oxide from the activated samples. Similarly, Santamaría *et al.*^46^ used a two stage carbonization technique to obtain a high surface area activated carbon (2700 m^2^ g^−1^) which resulted in high specific capacitance (250 F g^−1^). Mijowska *et al.*^82^ prepared porous carbon nanosheets (PCS) from PET waste and hybridized them with MnO_2_ nanoflakes resulting in PCS–MnO_2_ nanocomposite electrode which showed high specific capacitance of 210.5 F g^−1^ (at 0.5 A g^−1^ current density) which is lower than that obtained in the present study because of the low specific surface area of their material. On the other hand, Tang *et al.* and Chen *et al.* reported the preparation of high surface area carbon material from waste plastic material employing two-step carbonization and activation techniques. It can be observed that even with a substantially larger surface area, the performance of the activated carbon in 1 M Na_2_SO_4_ electrolyte is lower compared to that observed in the present study. It is worth noting that the synthesis process employed in our work is single step activation process using KOH as the degradation agent. The single step activation process resulted in a high surface area (1571 m^2^ g^−1^) with interconnected spherical flower-like carbon nanosheets where the high surface area carbon accounted for more active charge storage sites for the electrolyte ions, with the sheet-like morphology offered minimal resistance for easier ionic motion within the interconnected porous carbon framework during the charging/discharging process. From the results presented in this study, it can be concluded that the single stage activation process with KOH as activation agent, rather than utilizing complex catalysts, is an excellent room temperature approach to convert the PET plastic waste into value-added carbon materials for supercapacitor application. Since the ACF-900 sample exhibited the best electrochemical performance, its characteristics were further explored in a two-electrode device configuration for practical application.

**Table 1:** Comparison of specific capacitance of ACF 900 with other results reported in the literature

Precursor	SSA (m^2^ g^−1^)	*C*_s_ (F g^−1^)	Electrolyte	Ref
Polystyrene	2110	284.1 at 0.5 A g^−1^	6 M KOH	81
PCS–MnO_2_	453	210.5 at 0.5 A g^−1^	6 M KOH	82
Polystyrene	1051	149 at 0.5 A g^−1^	6 M KOH	85
Polyethylene (plastic bags)	1219	244 at 0.2 A g^−1^	EMIMBF_4_	86
Styrene acrylonitrile	1358	220 at 5 mV s^−1^	2 M KOH	73
Polystyrene foam	2700	250 at 0.05 A g^−1^	1 M H_2_SO_4_	46
Polystyrene foam	620	208 at 1 A g^−1^	6 M KOH	87
PP, PE, PS, PET, PVC	2198	137 at 0.2 A g^−1^	1 M Na_2_SO_4_	83
Poly(ethylene terephthalate)	2236	135 at 0.2 A g^−1^	1 M Na_2_SO_4_	84
PET (face shield)	1571	228 at 1 A g^−1^	1 M Na_2_SO_4_	This work

### 3.3 Electrochemical performance in two-electrode system

**Fig. 6:**
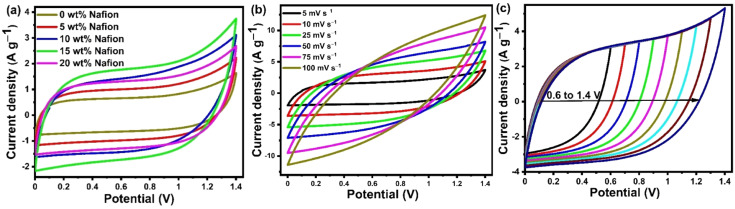
(a) CV graph at 5 mV s^−1^ scan rate of ACF-900 flexible supercapacitor device prepared with different binder conc. of Nafion. (b) CV at different scan rates. (c) CV at different potential rates at 10 mV s^−1^ scan rate for the supercapacitor device with 15 wt% Nafion loading.

Scalable slot-die coating was explored to fabricate large area supercapacitor electrodes by coating activated carbon ink onto stainless-steel mesh current collectors. The key parameters such as binder concentration and number of coatings have been systematically optimized to obtain good electrode coating and high-performance supercapacitors. Fig. 6 shows the electrochemical performance of the devices with electrodes containing different Nafion concentrations. The CV curves of the capacitors measured at 5 mV s^−1^ are given in Fig. 6(a). The curves clearly indicate that redox effects are absent in the potential range of 0 to 1.4 V, implying that the storage mechanism is entirely capacitive. Comparing the CV curves of the electrodes with different Nafion concentrations, it can be observed that the current density gradually increases with an increase in Nafion loading from 0 to 15 wt%, eventually reaching a maximum at 15 wt%, and thereafter decreases with 20 wt% Nafion loading. Upon comparing the CV curves at the same scan rate of 5 mV s^−1^, we find that the device with 15 wt% Nafion loading showed higher current response and a larger loop area indicating higher specific capacitance compared to the other devices. The improved current density for 15 wt% Nafion loading could be attributed to the formation of a crosslinking network in binder (Nafion)-based carbon electrodes which allows for efficient transport of electrolyte ions into/inside the porous network. This would lead to an increase in not only the electrical conductivity but also in the specific capacitance through coupling of the activated carbon with appropriate amounts of Nafion (15 wt% in this particular work). Fig. 6(b) shows the CV curves (at different scan rates) of the supercapacitor with 15 wt% Nafion loading. The CV curves are ‘rectangular’ at a modest scan rate (5–25 mV s^−1^), showing that the electrolyte can migrate through the micropores within a reasonable amount of time, indicating better charge/discharge reversibility. The degree of deviation of the CV curve from the rectangular shape is observed at higher scan rates (>25 mV s^−1^) because of the inadequate electrolyte movement at the electrode surface due to shorter reaction times.^56,88^ It can be observed that the current density increased with the increase in scan rate showing that the device with 15 wt% Nafion loading has an excellent rate capability. The CV curves of the 15 wt% Nafion loading supercapacitor in different potential windows at a scan rate of 10 mV s^−1^ is shown in Fig. 6(c). Even at 1.4 V, there is no discernible increase in anodic current density, showing that the built symmetric supercapacitor may be reversibly cycled and is stable across a wide voltage window.

**Fig. 7:**
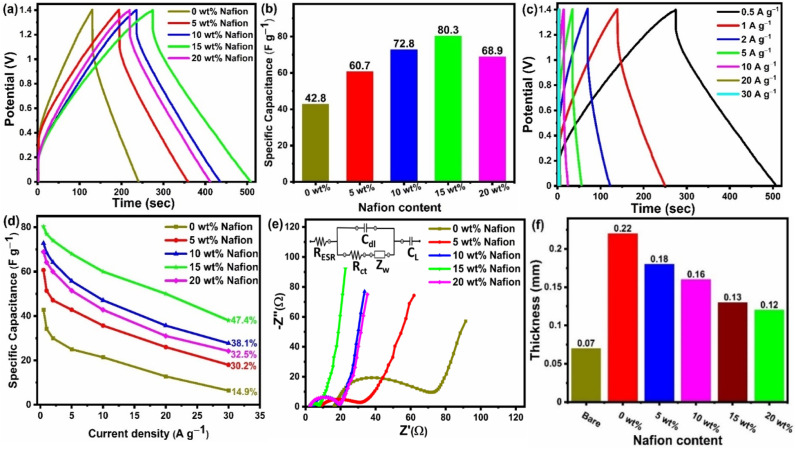
(a) GCD graph at 0.5 A g^−1^ current density of flexible supercapacitor device coated (using slot-die coater) with ACF-900 activated carbon electrodes with varying Nafion binder concentration. (b) Variation of specific capacitance (at 0.5 A g^−1^ current density) with respect to Nafion concentration. (c) GCD of the device with ACF-900 activated carbon and 15% Nafion coated electrodes measured at different current densities. (d) Specific capacitance as a function of current density of different ACF-900 devices with varying Nafion concentration. (e) Nyquist plots of ACF-900 flexible supercapacitor devices with inset showing the equivalent circuit fitting for the devices with different Nafion concentrations. (f) Thickness variation with Nafion content.

Galvanostatic charge discharge (GCD) analysis was performed in conjunction with the CV to provide a comprehensive understanding of the variation of specific capacitance w.r.t to the Nafion loading. The GCD profile of symmetric supercapacitors prepared with different binder (Nafion) concentrations are compared in Fig. 7(a) at a current density of 0.5 A g^−1^, and the specific capacitances of five devices are shown in Fig. 7(b). All the devices showed linear charge discharge (triangular shaped) behaviour within the 0 to 1.4 V potential window, but the GCD profile of the device with 15 wt% Nafion loading showed a longer discharge time, demonstrating higher specific capacitance as compared to other devices. According to the calculations from eqn (2), the 15 wt% Nafion loaded supercapacitor can deliver an excellent device specific capacitance of 80.3 F g^−1^ at a current density of 0.5 A g^−1^, which is significantly higher than for the other four devices. All of the measured GCD curves of 15 wt% Nafion loaded device at various current densities (over the current range of 0.5 to 30 A g^−1^) shown in Fig. 7(c) are triangular, demonstrating its exceptional capacitive reversibility at higher current densities. Moreover, the variation of specific capacitance at different current densities for the devices with 0–20% Nafion concentration (shown in Fig. 7(d)) reveal that the device with a 15 wt% Nafion concentration exhibits the highest capacitance retention (47.4%) and better electrochemical stability at higher current densities. The present study demonstrates the importance of optimizing the binder concentration during electrode fabrication to gain the best specific capacitance with outstanding capacitance retention.

To investigate the effect of Nafion concentration on the electrode kinetics, electrochemical impedance spectroscopy was carried out on the devices with varying concentrations of Nafion. As shown in Fig. 7(e), all the devices show a similar trend with a semicircle in the high frequency region and a capacitor-like response in the low frequency region. The intercept of the curve on the real axis indicates the resistance of the electrochemical system at high frequencies (*R*_ESR_, which is a combination of contact resistance at electrode/current collector interface, resistance of the electroactive material, contact resistance at the electrode/electrolyte interface, and ionic resistance of electrolyte). According to the fitting data, the corresponding *R*_ESR_ values for the electrodes coated with activated carbon containing 0 wt%, 5 wt%, 10 wt%, 15 wt%, and 20 wt% Nafion concentrations were 16.03, 5.45, 3.89, 2.58, and 2.28 Ω, respectively. One of the reasons for the decrease in *R*_ESR_ might be related to the thickness difference of the slot-die coated electrodes with different Nafion concentration [Fig. 7(f)]. Even though the number of coatings (five coatings in this work) was maintained constant, the as-prepared electrodes with different Nafion concentrations showed variable film thickness, which can be attributed to the porous amorphous carbon (ACF 900) dispersion in the inks. From Fig. 1(c) it can be observed that the electrodes prepared with 0 wt% and 5 wt% Nafion concentration had poor coating with high thickness variation, most likely due to the less dispersion of the amorphous carbon particles in the ink. On the other hand, electrodes with uniform coatings were obtained at higher Nafion concentration (≥10 wt%) due to less aggregation of amorphous carbon particles in the whole ink. Moreover, an increase in the Nafion concentration resulted in better adhesion between the current collector and the electrode film and also between the multiple carbon layers coated on top of each other, thereby decreasing the *R*_ESR_. The diameter of the semicircle in the high frequency region corresponds to the interfacial charge transfer resistance inside the porous framework. The corresponding charge transfer resistance (*R*_ct_) obtained after equivalent circuit fitting was found to be the highest (63.2 Ω) for the 0 wt% Nafion concentration and the lowest (5.28 Ω) value was obtained for a 15 wt% Nafion concentration. It can be observed that the charge transfer resistance decreases with the increase in Nafion content from 0 wt% to 15 wt% since an insufficient amount of binder (0 wt%) would result in a lower adhesion strength, making it difficult to form robust free-standing electrode films whereas increasing Nafion content to 15 wt% increases the ionic conductivity thereby enhancing the electrical conduction pathways within the porous electrode films. Further increasing the Nafion concentration to 20 wt% increases the *R*_ct_ value to 16.43 Ω because too high a Nafion concentration leads to the blocking of electrode pores. The EIS results are in agreement with the CV and GCD analysis and these findings suggest that the 15 wt% Nafion concentration in the ACF-900 ink improved charge transfer resistance due to the favourable electrode–electrolyte contact and also the cross-linking inside the porous carbon framework. The impedance spectrum of the corresponding devices with different Nafion concentrations was fitted with five components, as shown in the inset of Fig. 7(e): series resistance (*R*_ESR_), charge transfer resistance (*R*_ct_), Warburg impedance related to the electrolyte ion diffusion (*Z*_w_), double layer capacitance (*C*_dl_) and mass capacitance (*C*_L_).^89^ At high frequencies, the *R*_ct_–*C*_dl_ circuit is responsible for the transfer of ions between the micro/mesopores of the electrode material, whereas *Z*_w_ is responsible for the diffusion of electrolyte ions in the middle frequency region. At low frequencies, the nearly straight line indicates the capacitive behavior of the device which is represented by *C*_L_ in the equivalent circuit.

**Fig. 8:**
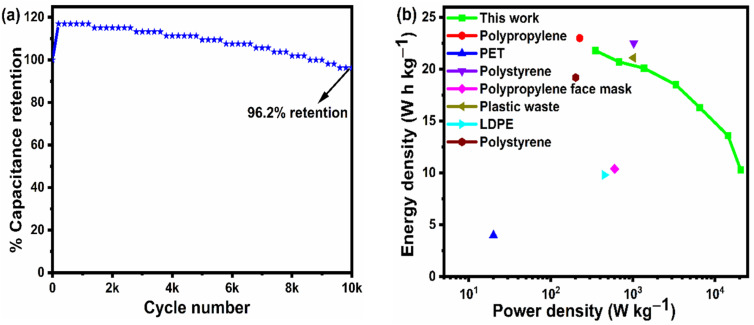
(a) Cyclic stability of 15 wt% Nafion loaded supercapacitor performed over 10 000 cycles at 2 A g^−1^ current density. (b) Ragone plot of 15 wt% Nafion loaded device compared with other supercapacitors based on carbon derived from plastic waste reported in literature.

**Fig. 9:**
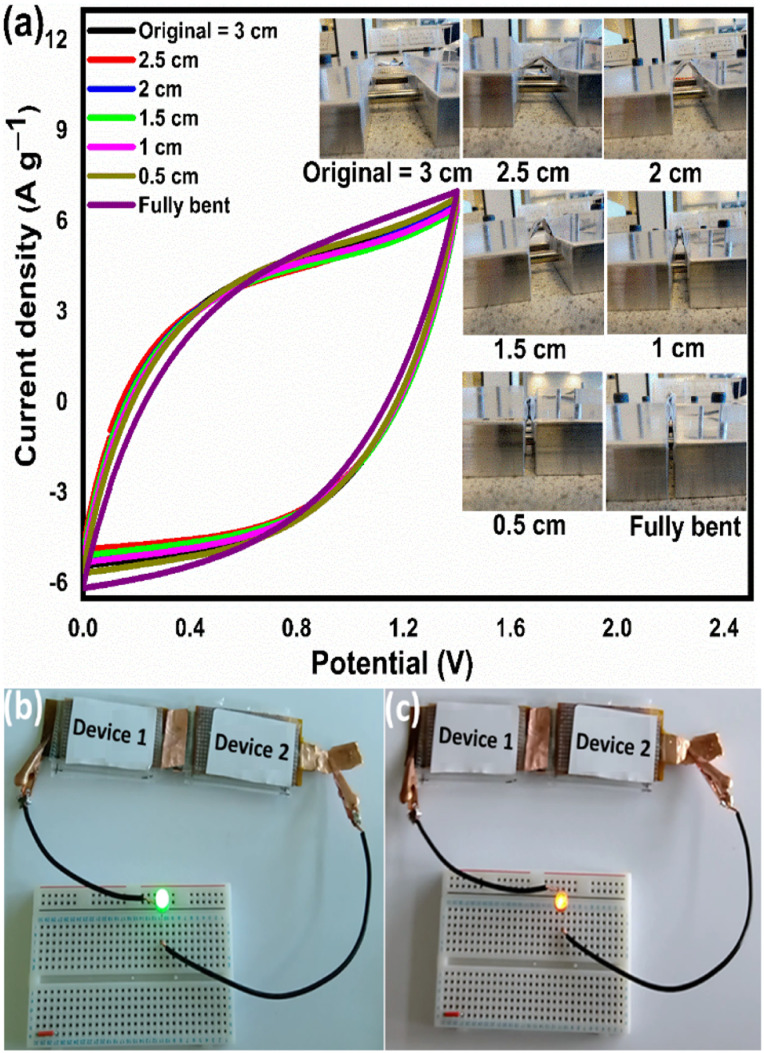
(a) CV curves of the flexible supercapacitor at different bending radii (length mentioned is the distance between the clamps) measured at a scan rate of 25 mV s^−1^. (b) Digital photograph of the two devices connected in series powering a green LED. (c) Digital photograph of the two devices connected in series powering a red LED.

From the above analysis, it was concluded that the device with 15 wt% Nafion concentration showed excellent electrochemical performance. The device was further tested for cyclic stability. The cycle stability of the supercapacitor is a critical evaluation criterion for practical applications. Fig. 8(a) shows the electrochemical cyclic stability of the flexible supercapacitor device performed over 10 000 cycles at 2 A g^−1^ current density. As the number of GCD cycles increases, the specific capacitance increases slightly at first and then there is a gradual decrease. During the first 1200 cycles of the GCD scan, the percentage capacity retention was observed to improve from 100 to 116.9 percent. With increased GCD cycles, the capacitance may rise due to the progressive activation of the surface, allowing easier access of the electrolyte ions in the micropores of the carbonized face shields. However, there is a gradual and steady decrease in the specific capacitance after 1200 cycles and 15 wt% Nafion loaded device finally retained 96.2% of its initial capacitance, revealing a prominent long-term cycle stability. The reason for the decrease in the specific capacitance might be attributed to the sheet like morphology in the porous matrix, which tend to stack during the continuous charge discharge cycles. Overall, the outstanding electrochemical performance and cycle stability imply that this flexible supercapacitor could be one of the potential energy storage devices of the future. The electrochemical performance of the flexible supercapacitor device with 15 wt% Nafion concentration was further evaluated using a Ragone plot by calculating energy and power densities from the discharge curves using eqn (4) & (5). As shown in Fig. 8(b), at a power density of 348.8 W kg^−1^ the fabricated device exhibited a high energy density of 21.8 W h kg^−1^ which was comparable to and even higher than the values reported for supercapacitors based on other plastic derived activated carbon electrodes prepared with different fabrication techniques reported in the literature.^47,53–55,87,90^ Owing to its good rate capability, at current density of 30 A g^−1^, the flexible device still achieved an energy density of ∼47% of its maximum, even at high power density of 20.6 kW kg^−1^. This is an attractive and significant result compared to most recently reported devices with plastic derived carbon electrodes. The materials and coating method described here can be used to produce high-performance supercapacitor electrodes in a cost-effective, efficient, and scalable manner. Besides the excellent electrochemical performance at normal stage of operation, the ability to withstand deformation (such as bending) without significant loss in the performance is an important characteristic of flexible supercapacitor device. By clamping the 15 wt% Nafion loaded device to a linear stretcher [a clamping system with one end fixed and the other end is movable], as illustrated in the inset of Fig. 9(a), the mechanical stability of the device was examined. The movable end of the clamp was shifted in steps of 0.5 cm towards the fixed end and the CV measurements were taken each position. As shown in Fig. 9(a), the CV curves remained quasi-rectangular at different bending positions without any significant deviation in its behavior indicating that the bending has minimum/no impact on the electrochemical performance of the device. These results imply that the flexible supercapacitor has excellent mechanical and electrochemical stability during different bending radii, indicating a huge promise for flexible and portable energy storage devices. To further evaluate the real time application of the 15 wt% Nafion loaded device, two symmetric supercapacitors were connected in series as shown in Fig. 9(b) and (c). These supercapacitor devices were charged using a regulated power supply and discharged using green and red LED lights. Fig. 9(b) and (c) shows a photograph of the devices connected in series, lighting up red and green LEDs suggesting the potential applicability of the slot-die coated flexible supercapacitors.

## 4. Conclusions

To conclude, we have demonstrated a cost-effective one-step strategy to synthesize activated carbon consisting of spherical flower like carbon nanosheets and amorphous porous flakes from upcycling waste PET face shield. N_2_ adsorption isotherms and pore size distribution analysis clearly indicated that the specific surface area and pore volume can be tuned by adjusting the activation temperature. The material exhibited a high specific surface area of 1571 m^2^ g^−1^ under optimal activation temperature of 900 °C with mesopores centred between 3–5 nm. As supercapacitor electrodes, the obtained activated carbon samples showed excellent capacitive performance in three-electrode configuration with a highest specific capacitance of 228.2 F g^−1^ at 1 A g^−1^ current density, low *R*_ct_ and good rate capability in 1 M Na_2_SO_4_ electrolyte. Secondly, we demonstrated slot-die coating as a potential scalable technique for production of nanoporous activated carbon electrodes for flexible supercapacitors by using activated carbon inks. Moreover, we studied the effect of the binder (Nafion) concentration on the overall performance of flexible supercapacitors. It was observed that the slot-die coated activated carbon electrodes with 15 wt% Nafion concentration showed the best electrochemical performance. The optimized activated carbon with 15 wt% Nafion concentration based flexible supercapacitor demonstrated highest energy density of 21.8 W h kg^−1^ and highest power density 20 600 W kg^−1^ and displayed outstanding cyclic stability of 96.2% after 10 000 cycles at 2 A g^−1^ current density. Thus our devised method for dealing with COVID19 PET face shield plastic wastes holds a lot of promise for real-world applications. The findings suggest that simple, low-cost, large-scale slot-die coating can be used to manufacture large-area activated carbon electrodes for supercapacitors, especially showing great potential applications in commercialisation of activated carbon-based flexible supercapacitors.

## Author contributions

RKKR designed, carried out and analysed most of the practical work and drafted the manuscript. AC carried out Raman measurements. LŠ carried out the XPS measurements. AI conceptualised and directly supervised the work and helped in results interpretation. All the authors provided inputs to the manuscript.

## Conflicts of interest

There are no conflicts of interest to declare.

## Supplementary Material

RA-014-D2RA06809E-s001
