# Supplementary material for: Scalable slot-die coated flexible supercapacitors from upcycled PET face shields
Source: RSC Adv. 2024 Apr 19;14(18):12781–95. doi: 10.1039/d2ra06809e (PMC11027888; doi:10.1039/d2ra06809e)
Supplement: RA-014-D2RA06809E-s001 [file RA-014-D2RA06809E-s001.pdf]

Electronic Supplementary Material (ESI) for RSC Advances.
This journal is © The Royal Society of Chemistry 2022

Supporting Information

Scalable Slot-die Coated Flexible Supercapacitors from Upcycled PET face shields

R. Kiran Kumar Reddy[a], Andrew Callander[b], Lidija Šiller[c], Karen Faulds[b], Leonard Berlouis[a], and Aruna Ivaturi[a*]

[a]Smart Materials Research and Device Technology (SMaRDT) Group, Department of Pure and Applied Chemistry, University of Strathclyde, Thomas Graham Building, Glasgow, G1 1XL, UK
[b]Centre for Molecular Nanometrology, Department of Pure and Applied Chemistry, University of Strathclyde, Technology Innovation Centre, 99 George Street, Glasgow, G1 1RD, UK
[c]Newcastle University, School of Engineering, Newcastle upon Tyne, NE1 7RU, UK

Corresponding author: email aruna.ivaturi@strath.ac.uk

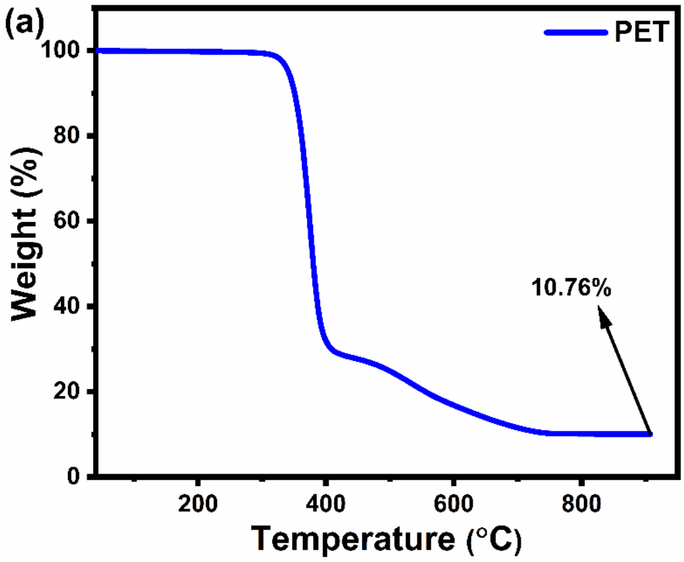

Fig. S1 (a) TGA thermogram of face shield (PET) pieces

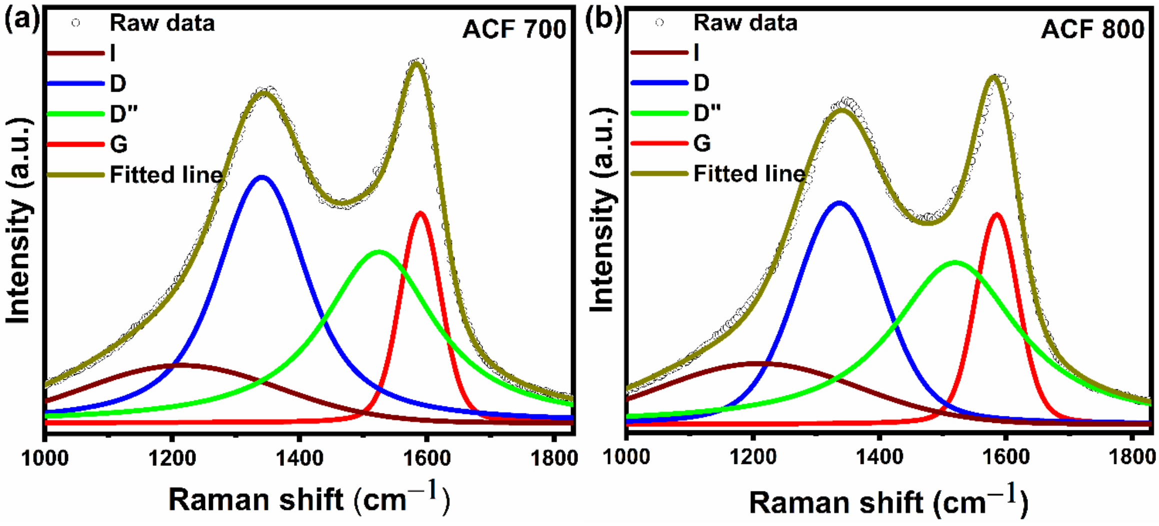

Fig. S2 Deconvoluted Raman spectra of (a) ACF-700 (b) ACF-800 samples

Table S1: Parameters employed for peak deconvolution

Sample	Peak Type	Center	Height	Area	FWHM	A_D/A_G	I_D/I_G
ACF-700	I	1215.26	517.76	198658	360.25	3.18	1.17
	D	1341.01	2217.94	532301	168.8		
	D"	1524.76	1542.82	511002	212.38		
	G	1589.84	1891.53	167142	76.31		
ACF-800	I	1210.3	730.04	287962	370.52	2.26	1.04
	D	1336.6	2661.31	520355	164.41		
	D"	1519.76	1944.12	721544	239.92		
	G	1585.46	2522.5	233720	63.24		
ACF-900	I	1210.78	472.64	168899	322.58	1.84	0.81
	D	1340.41	2921.62	598043	130.31		
	D"	1517.96	898.85	266487	188.76		
	G	1577.07	3594.27	388035	36.68		

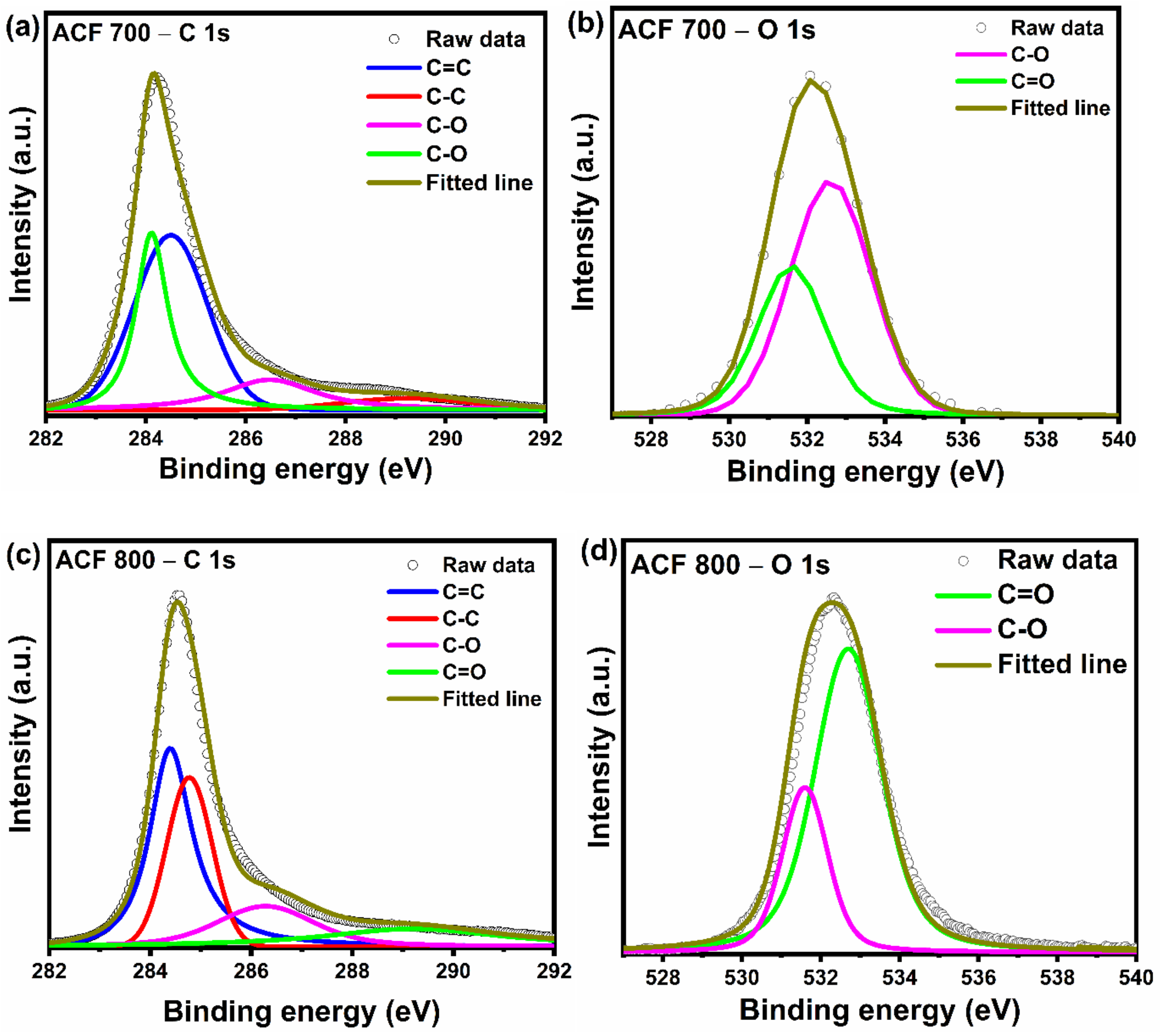

Fig. S3 (a) Deconvoluted C1s spectra of ACF-700 sample (b) Deconvoluted O1s spectra of ACF-700 sample (c) Deconvoluted C1s spectra of ACF-700 sample (d) Deconvoluted O1s spectra of ACF-700 sample

Table S2: The C and O contents of all samples from XPS analysis.

**Sample**	**C (wt%)**	**O (wt%)**
ACF-700	75.87	24.13
ACF-800	85.81	14.19
ACf-900	94.64	5.36

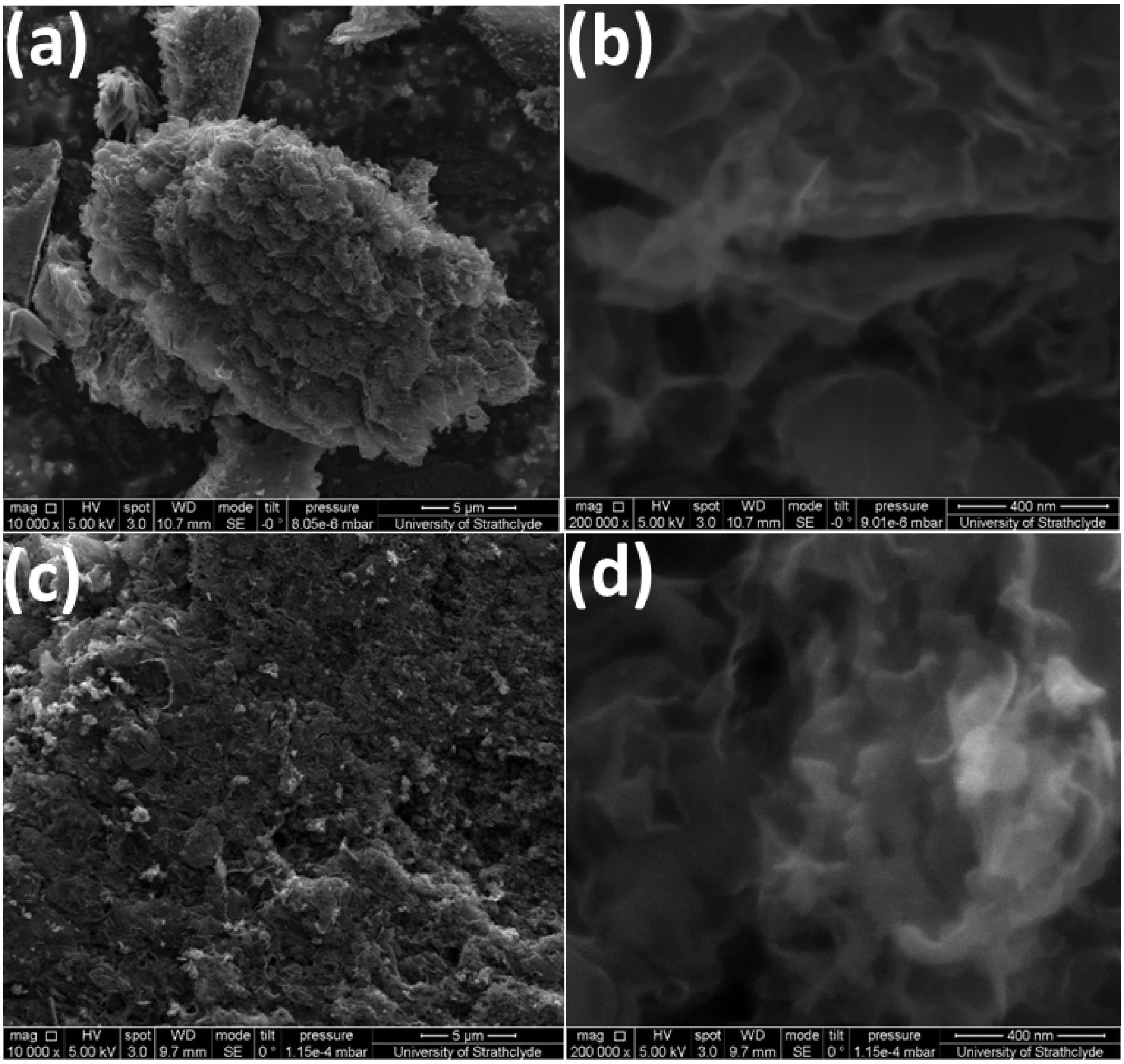

Fig. S4 (a - b) FESEM images of ACF-700 sample (c - d) FESEM images of ACF-800 samples

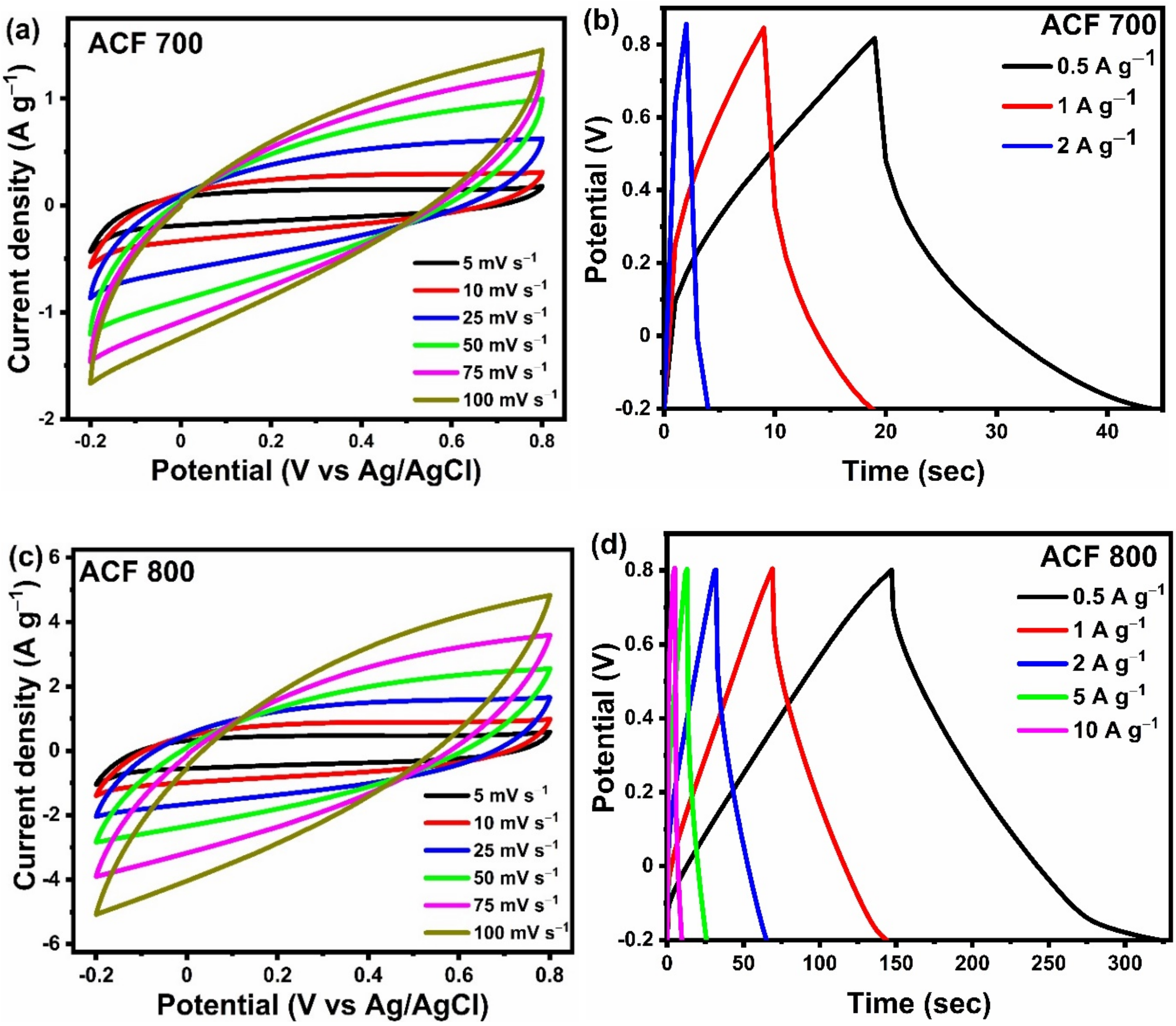
(a)
ACF 700
Current density (A g^{-1})
Potential (V vs Ag/AgCl)
5 mV s^{-1}
10 mV s^{-1}
25 mV s^{-1}
50 mV s^{-1}
75 mV s^{-1}
100 mV s^{-1}
(b)
ACF 700
0.5 A g^{-1}
1 A g^{-1}
2 A g^{-1}
Potential (V)
Time (sec)
(c)
ACF 800
Current density (A g^{-1})
Potential (V vs Ag/AgCl)
5 mV s^{-1}
10 mV s^{-1}
25 mV s^{-1}
50 mV s^{-1}
75 mV s^{-1}
100 mV s^{-1}
(d)
ACF 800
0.5 A g^{-1}
1 A g^{-1}
2 A g^{-1}
5 A g^{-1}
10 A g^{-1}
Potential (V)
Time (sec)

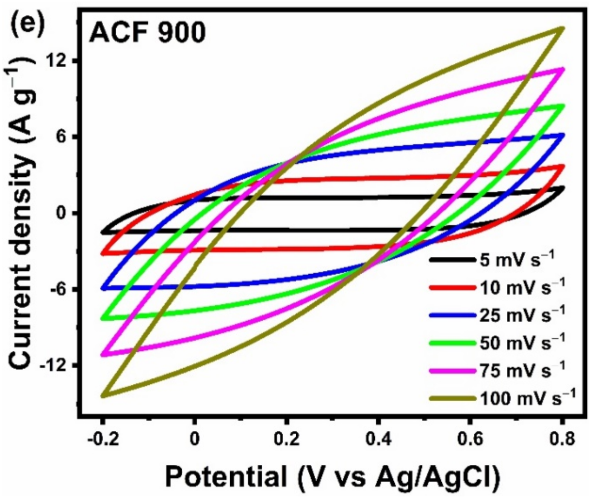

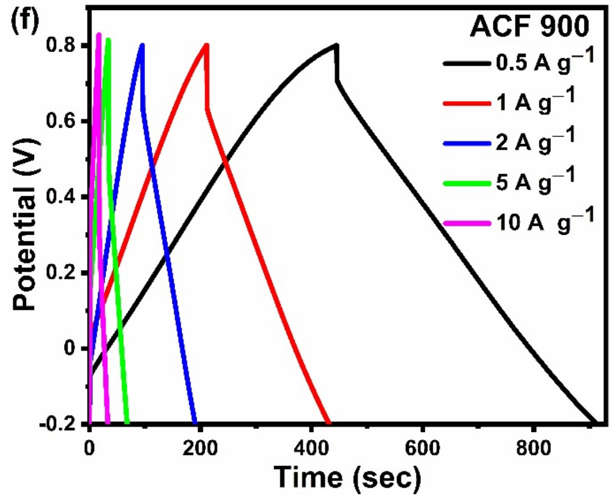

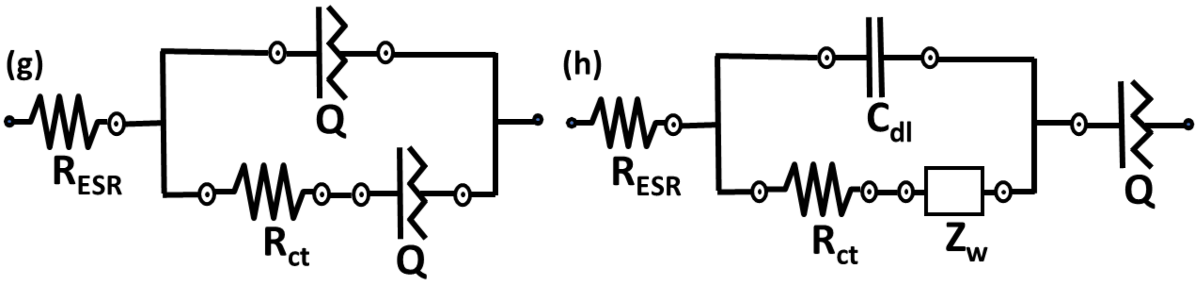

Fig. S5 (a & b) CV graph at different scan rates and GCD at different current densities for ACF-700 electrode (c & d) CV graph at different scan rates and GCD at different current densities for ACF-800 electrode (e & f) CV graph at different scan rates and GCD at different current densities for ACF-900 electrode tested in three-electrode configuration in 1M Na_2SO_4 electrolyte (g) Equivalent circuit model of ACF-700 (h) Equivalent circuit model of ACF-800
